# Origin of Retinal Oscillatory Potentials in the Mouse, a Tool to Specifically Locate Retinal Damage

**DOI:** 10.3390/ijms24043126

**Published:** 2023-02-04

**Authors:** Fei Liao, Haitao Liu, Santiago Milla-Navarro, Pedro de la Villa, Francisco Germain

**Affiliations:** 1 Department of Systems Biology, University of Alcalá, 28871 Alcalá de Henares, Spain; 2 IRYCIS, Hospital Ramón y Cajal, 28034 Madrid, Spain

**Keywords:** oscillatory potentials, bipolar cells, neurotransmitter, retina, mice

## Abstract

To determine the origin of oscillatory potentials (OPs), binocular electroretinogram (ERG) recordings were performed under light and dark adaptation on adult healthy C57BL/6J mice. In the experimental group, 1 μL of PBS was injected into the left eye, while the right eye was injected with 1 μL of PBS containing different agents: APB, GABA, Bicuculline, TPMPA, Glutamate, DNQX, Glycine, Strychnine, or HEPES. The OP response depends on the type of photoreceptors involved, showing their maximum response amplitude in the ERG induced by mixed rod/cone stimulation. The oscillatory components of the OPs were affected by the injected agents, with some drugs inducing the complete abolition of oscillations (APB, GABA, Glutamate, or DNQX), whereas other drugs merely reduced the oscillatory amplitudes (Bicuculline, Glycine, Strychnine, or HEPES) or did not even affect the oscillations (TPMPA). Assuming that rod bipolar cells (RBC) express metabotropic Glutamate receptors, GABA_A_, GABA_C_, and Glycine receptors and that they release glutamate mainly on Glycinergic AII amacrine cells and GABAergic A17 amacrine cells, which are differently affected by the mentioned drugs, we propose that RBC-AII/A17 reciprocal synapses are responsible for the OP generation in the ERG recordings in the mice. We conclude that the reciprocal synapses between RBC and AII/A17 are the basis of the ERG OP oscillations of the light response, and this fact must be taken into consideration in any ERG test that shows a decrease in the OPs’ amplitude.

## 1. Introduction

Knowing precisely the retinal neurons affected in the course of an ophthalmological disease is essential to be able to establish its diagnosis, prognosis, and treatment. In the course of a neurodegenerative disease, we can find different functional responses that can be recorded using electroretinographic techniques. In this way, oscillatory potentials (OP) are characterized by being a high-frequency, low-amplitude response, which is superimposed on the rising phase of the b-wave of the full-field flash electroretinogram (ERG) (review: [1]). It is believed that the b wave elicited by low-intensity light flashes, recorded under scotopic conditions, originates exclusively from rod bipolar cells, while the b wave elicited by high-intensity light flashes originates from rod bipolar cells and ON and OFF cone bipolar cells; under photopic conditions, the b wave is originated from ON and OFF cone bipolar cells [2]. The OPs were first described in 1937 in the frog ERG [3], but its exact genesis is still unknown. It has been discussed if OPs can be considered biomarkers for neuronal function and if they may be useful for the diagnosis of eye diseases, such as diabetic retinopathy [4,5,6,7], Bothnian retinal dystrophy [8], myopia [9], high intraocular pressure [10], and various types of retinal degeneration [11,12]; hence, the importance of knowing their origin and mechanism of genesis.

**Figure 1:**
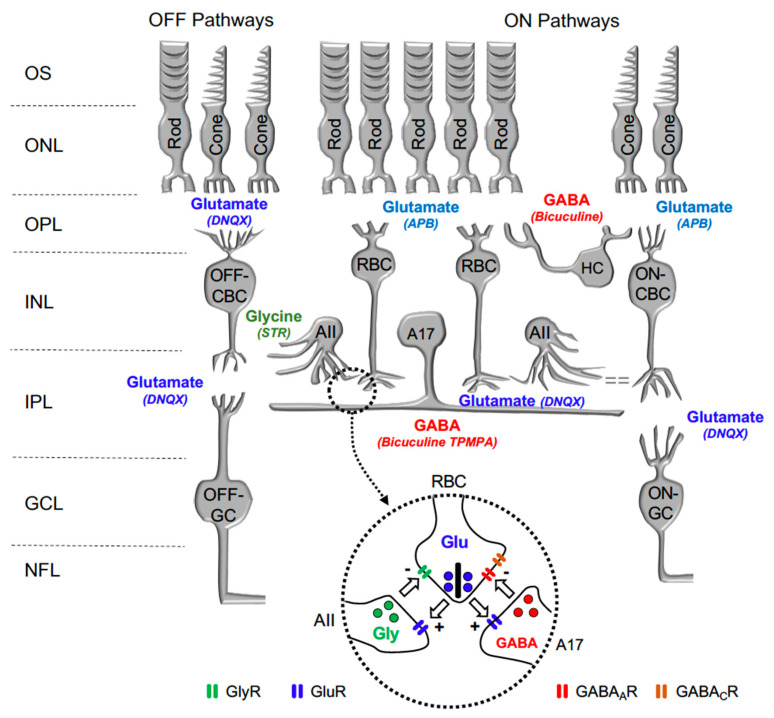
Diagram illustrating the main neurotransmitters and membrane receptors expressed on retinal cells. In the retina, Glutamate (Glu) released from photoreceptors (rods and cones) acts on APB-sensitive metabotropic glutamate receptor type 6 (mGluR6) expressed by ON-type Rod Bipolar Cells (RBC) and Cone Bipolar Cells (CBC) and DNQX-sensitive ionotropic receptor expressed by OFF-type CBC. Glutamate released from CBC act on DNQX-sensitive ionotropic receptor expressed by Ganglion cells (GC). Glutamate released from RBC act on DNQX-sensitive ionotropic receptor expressed by AII, and A17 amacrine cells. GABA released by Horizontal cells (HC) and A17 Amacrine cells acts on Bicuculine and TPMPA-sensitive ionotropic GABA_A_ and GABA_C_ receptors expressed by RBC. Glycine released by AII Amacrine cell acts on Strychnine (STR) sensitive ionotropic receptor expressed by RBC and OFF-CBC. OS: outer segments of photoreceptors; ONL: outer nuclear layer; OPL: outer plexiform layer; INL: inner nuclear layer; IPL: inner plexiform layer; and GCL: ganglion cells layer. *Inset*: detail of the reciprocal synapse between RBC, AII, and A17 Amacrine cells. GlyR: Glycine Receptor; GluR: Glutamate Receptor; GABA_A_R: GABA_A_ Receptor; GABA_C_R: GABA_C_ Receptor.

In the retina, visual transmission is divided into ON and OFF pathways, as glutamate continuously released by photoreceptors in the dark hyperpolarizes ON bipolar cells (BC) and depolarizes OFF-BC, due to the expression of different glutamate receptors at their dendrites. While ON-BC contain inhibitory metabotropic glutamate receptor type 6 (mGluR6), OFF-BC contain excitatory AMPA/KA glutamate ionotropic receptors [13]. Retinal interneurons such as horizontal cells and amacrine cells also play a significant function in lateral interaction at the outer plexiform and inner plexiform layers. Both horizontal cells and amacrine cells may release GABA or Glycine on GABAergic or glycinergic membrane receptors expressed by rod bipolar cells and cone bipolar cells. Figure 1 shows a scheme of the mouse retina, including the main retinal neurotransmitters related to our work and their membrane receptors.

It has been suggested that OPs were dependent on the retinal circuitry and that the ON pathway seemed to play a critical role in OP generation, as its response was severely decreased after blocking synaptic transmission between photoreceptors and ON-type rod BC [14]. However, it is not completely accepted that the ON-BC themselves make any direct critical contribution to the generation of OPs [15,16,17].

Neurodevelopmental and neuropharmacological studies suggested that OPs reflect the activity of multiple generators in the proximal retina that could involve BC, amacrine cells, and/or ganglion cells [1,18,19]. Currently, the physiological origin of OPs remains under debate, which hampers their potential clinical use and also makes it difficult to understand normal retinal function. In this work, mice have been used due to their great importance as a model for the study of retinal diseases, their clear OP response, and they have a simple peak frequency higher than that of other animals studied [20]. The rod and cone visual pathways have been segregated in order to examine the contribution of the different neural mechanisms of the retina to the components of the OPs. For this purpose, a series of chemical agents has been injected into the mouse eye, and ERG OPs have been recorded.

## 2. Results

### 2.1. The OP Components of the Control Response

To verify that the mere intravitreal injection had no effect on the recordings, in the first series of experiments (*n* = 6 animals), PBS was injected into the right eye, while no injection was made into the left eye. The OP recordings show a series of positive and negative oscillating components (Figure 1, top recording). The number of components of the OPs that could be measured varied as a function of the intensity of the light flashes, the adaptation state, and the electrical filters used during the recording procedure. Under our experimental conditions, there were six positive and negative peaks (or components) that appeared alternately and could be clearly identified under scotopic (rod-driven and mixed rod/cone-driven) conditions. No statistically significant differences were observed in the amplitude of the OP response when comparing both eyes. It can be stated that the manipulation involved in the intravitreal injection does not modify the OP amplitudes during recordings.

#### Waveforms

The OP waveform changed considerably depending on the light stimuli to which the retina was subjected, indicating that it is susceptible to the different proportions of photoreceptors (rods and cones) that are stimulated by light. In the rod-driven responses, most OPs showed small ripples, which may be the result of rapid low-amplitude adaptive electrical signals. On the other hand, the OP waveforms in the mixed rod/cone-driven responses, either the positive or negative waves, showed a high amplitude (ca. 100 μV), so each component was easy to identify. In the mouse, the OP responses driven only by cones were almost not distinguished from the recording noise.

### 2.2. The OPs in Mixed Rod/Cone-Driven Response under the Effect of Injected Agents

**Figure 2:**
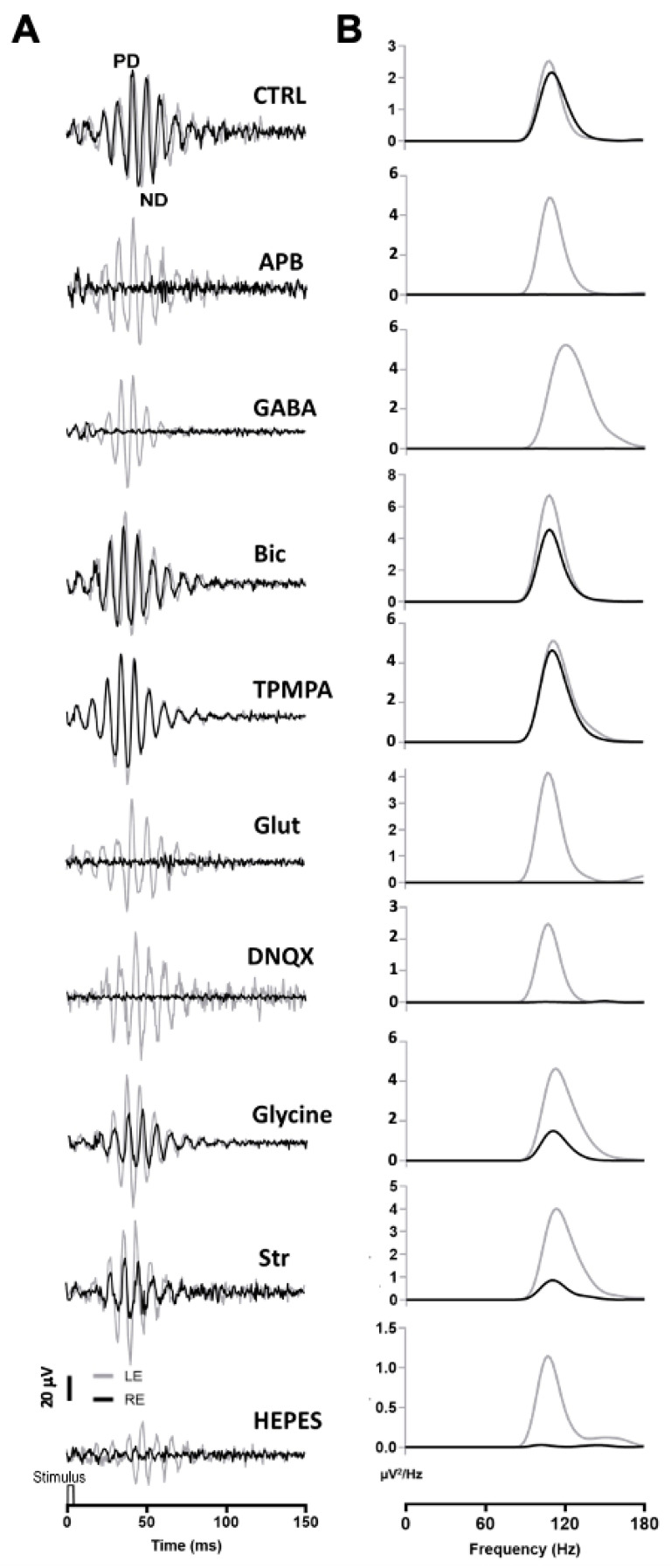
Oscillatory potentials (OP) recorded after intraocular injection of agents: (**A**) Representative recording of mixed rod/cone-driven OP under dark adaptation in response to light stimuli of 15 cd·s·m^−2^ in control conditions and under the effect of various agents. All recordings were obtained from an animal sample of the different experimental conditions. High-pass digital filtering of 100 Hz was performed on ERG flash recordings. The right eye (RE, black line) was injected with 1 μL of PBS solution (CTRL) or 1 μL of PBS solution containing APB (25 mM), GABA (100 mM), Bicuculline (10 mM), TPMPA (25 mM), Glutamate, DNQX (30 mM), Glycine (10 mM), Strychnine (25 mM), or HEPES (25 mM). One μL of PBS was injected into the left eye (LE, grey line). (**B**). Power Density (μV^2^/Hz) analysis was performed on trace recordings shown in A. Analysis of the right eye (black line) is superimposed on the left eye (grey line).

A series of agents were injected into the right eye and ERG responses were recorded. In these experiments, 1 μL of PBS was injected into the left eye, while the right eye was injected with 1 μL of PBS containing the different agents. All recordings were performed simultaneously from both eyes. Under scotopic conditions, the mixed rod/cone-driven OP waveform was robust and each OP component was easy to identify (Figure 2A). In order to effectively analyze the effect of different agents on ERG OP, high-pass digital filtering was applied to the recorded traces, and Fast Fourier Transform was applied to each 100 Hz filtered recording trace (Figure 2B). The power density (μV^2^/Hz) representations of OPs show a maximum peak of around 110–120 Hz in all recordings obtained from control eyes (Figure 2B), while the effect of the different agents is shown as a decrease in the power density peak.

After intravitreal injection of APB (mGluR6 receptor agonist), the OPs were almost abolished in the mixed rod/cone-driven response. The application of GABA selectively eliminated most of the oscillations. However, the injection of Bicuculline (GABA_A_ receptor antagonist) did induce a reduction in the OP response amplitude, but did not abolish the oscillations. We further injected TPMPA (GABA_C_ receptor antagonist), and almost no effect on OP response amplitude was observed. The intraocular injection of Glutamate or DNQX (AMPA/KA receptor antagonist), selectively eliminated the oscillations of the OP. When Glycine was injected into the right eye, no effect on the frequency of OP was observed; although, the amplitude of the oscillations decreased significantly. We further injected Strychnine (a Glycine receptor antagonist), and a decrease in OP response amplitude was observed. Finally, in order to test if pH changes could affect the OP oscillations, HEPES was injected into the right eye. A decrease in the OP response amplitude could be observed. The peak amplitude of the power density analyses was averaged from a series of experimental animals injected with the above-mentioned agents. Table 1 shows the average data of the power density peak amplitudes from ERG recordings from the control right eye compared with those from injected left eye, after digital filtering of oscillating components. Significant differences are shown between control left eyes and drug-injected right eyes.

### 2.3. The OPs in Rod-Driven and Cone-Driven Response under the Effect of Injected Agents

**Figure 3:**
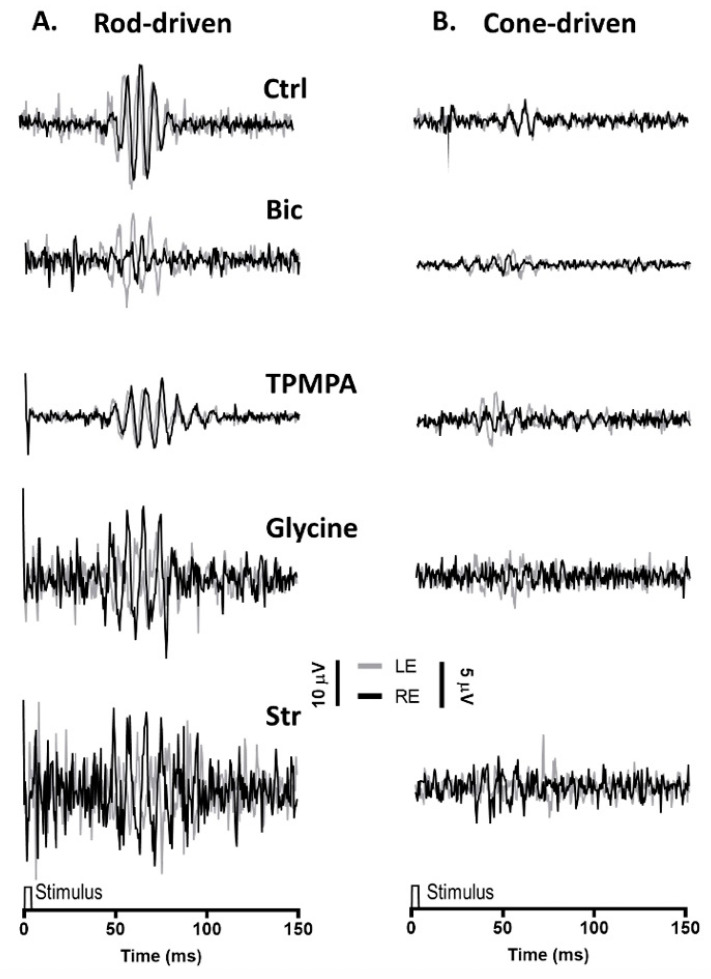
Oscillatory potentials (OP) driven by rod and cone photoreceptors after intravitreal injections of agents: (**A**), Representative rod-driven OP recordings under dark adaptation in response to light stimuli of 0.01 cd·s·m^−2^ and (**B**) cone-driven OP recordings under light adaptation in response to light stimuli of 15 cd·s·m^−2^. Control recording (Ctrl) and the effect of Bicuculline (10 mM), TPMPA (25 mM), Glycine (10 mM), and Strychnine (10 mM) is shown. All pairs of recordings were obtained from an animal sample of the different experimental conditions.

Similar to the mixed rod/cone-driven OP, the same series of agents were injected into the right eye and rod-driven OP responses (Figure 3A) were recorded simultaneously from both eyes. The application of APB, GABA Glutamate, and DNQX selectively eliminated all oscillations. While the injection of Bicuculline did induce a reduction in the OP response amplitude, without abolishing the oscillations, the injection of TPMPA did not affect the OP responses. Finally, when Glycine or Strychnine was injected into the right eye, the amplitude of the rod-driven oscillations decreases significantly. Cone-driven OP responses (Figure 3B) were almost not distinguished from the recording noise.

## 3. Discussion

With the intention of determining the cellular origin of the ERG oscillations of the OP responses, and their possible clinical applications in the evaluation of retinal damage, this work analyzed the modification of OPs after intravitreal injection of different agents, agonists, and antagonists of retinal neurotransmitters.

The OPs recorded under control conditions show a series of positive and negative components. The number of peak components observed in the rod-driven (scotopic condition) and in cone-driven (photopic condition) responses were smaller than those of the mixed rod/cone response (under scotopic condition). The mixed rod/cone-driven response exhibited the most robust and highest amplitude of the OP waveforms. These results suggest that a mixture of retinal neurons is involved in the genesis of OPs, which reflect more the retinal kinetics of the inner plexiform layer, rather than that of a specific cell type (review: [1]). Thus, the observed differences could be the result of the operation of different visual circuits in which the different response of the OPs would be based on the contribution of cells in the rod visual pathway [2,21]; although, the contribution of the retinal cone visual pathway must not be discarded (see below).

The intraocular application of agents has provided some clues about the mechanism that generates the scotopic-recorded OPs. With the intention of explaining these mechanisms more clearly, the sites of action of the neuroactive agents used in this study are shown in Figure 1. After intraocular injection of TPMPA, no effect on OP oscillation amplitudes could be observed. On the contrary, Bicuculline, Glycine, Strychnine, and HEPES, induced a significant decrease in the OP amplitudes. However, all the other agents (APB, GABA, Glutamate, and DNQX) almost completely eliminate the oscillations of the OPs. These effects indicate that glycinergic and GABA_A_ receptors are critical for the genesis of OPs, but the contribution of the GABA_C_ receptor must not be considered. Some studies have previously described in different animal species that both GABA_A_ and Glycine receptor antagonists and agonists can partially or completely eliminate OPs: carp and mudppupy [1]; rat: [22,23,24]; and mice: [25,26]. In our experiments, we used a single effective drug concentration; although, the use of different drug concentrations may also justify the effects observed by other authors, since the effect of the GABA_A_ and Glycine receptors depend on the concentration of the applied agents (review: [27,28]).

**Figure 4:**
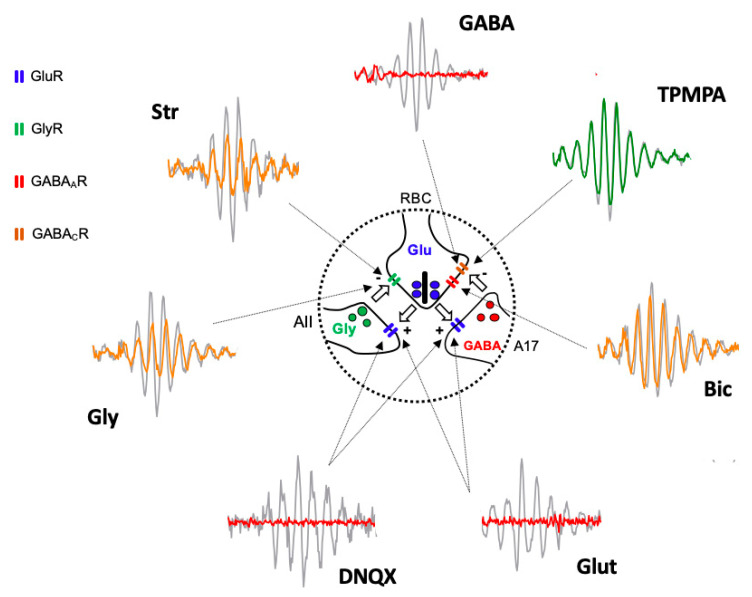
Diagram illustrating the site of action of injected agents generating Oscillatory Potentials. Schematic representation of the reciprocal excitatory/inhibitory synapses between Rod Bipolar Cell (RBC) axon terminal and AII, and A17 amacrine cells extracted from Figure 1 and Oscillatory Potential traces extracted from Figure 2 shows the site of action (arrows) of injected drugs and the effect on Oscillatory Potentials. Grey traces correspond PBS injected eyes and colored traces correspond to drug-injected eyes (red = abolishing OP; yellow = partially reducing OP; green = not affecting OP). Intravitreally injected Glutamate (Glut) and DNQX act on GluR expressed by AII and A17 cell processes. GABA, Bicuculline (Bic), and TPMPA act on GABA_A_R and GABA_C_R expressed by RBC axon terminal. Glycine (Gly) and Strychnine (Str) act on GlyR expressed by RBC axon terminal. AII: type II amacrine cell; A17: type 17 amacrine cell; GlyR: Glycine Receptor; GluR: Glutamate Receptor; GABA_A_R: GABA_A_ Receptor; GABA_C_R: GABA_C_ Receptor.

Based on the obtained results, we suggest that the oscillatory response is generated in the synapse that takes place between the axon terminals of the rod bipolar cells and the amacrine cells (Figure 4). The effects of Glutamate, and its agonist APB, on mGluR6 receptors, are easily explained based on Figure 1, since the continuous activation of mGluR6 receptors by APB does not allow ON-type bipolar cells to detect the decrease in glutamate release by the axon terminals of the photoreceptors; therefore, APB completely abolished the ON-type bipolar cell response [29] and, therefore, the oscillatory responses. The effect observed by the intraocular injection of Glutamate or its antagonist DNQX on ionotropic receptors is also easily explained based on Figure 1 and Figure 4. Both the glycinergic AII and GABAergic A17 amacrine cells postsynaptic to rod bipolar cells [30,31,32] would be continuously stimulated (due to the effect of Glutamate) or inhibited (due to the effect of DNQX), producing, in both cases, the abolition of Glycine or GABA release and, therefore, the oscillatory responses. The effect of GABA on the oscillatory response is also easily justified, since rod bipolar cells express GABA_C_ receptors at their axon terminal [33,34], as shown in Figure 4.

The GABA activation of the GABA_C_ receptors, which are more sensitive than GABA_A_ receptors, shows an almost inexistent temporal adaptation and produces the complete inhibition of the rod bipolar cells, which would stop releasing glutamate from their axon terminal; therefore, any oscillatory response would disappear. This fact would also justify why TPMPA does not produce any effect, since the blockade of these receptors does not influence the ability of rod bipolar cells to release glutamate, nor does it modify their sensitivity to Glycine released by AII amacrine cells or GABA released from A17 amacrine cells. The effect of Bicuculline could be explained by the blockade of GABA_A_ receptors expressed in rod bipolar cells, either by those expressed at the dendritic level or by those located at the axon terminal. Although the blockade of GABA_A_ receptors in the axon terminal could partially explain the decrease in the amplitude of the oscillations (Figure 4), the reciprocal synapse between rod bipolar cells and AII amacrine cells would still be present, which would justify the oscillatory responses being partially maintained. Another explanation for the observed effect of Bicuculline is that it could act on the GABA_A_ receptors expressed by the horizontal cells [35,36,37,38], and based on the feedback they exert on the photoreceptors, modify the release of Glutamate by the axon terminals of the rods. The same effect could be caused by HEPES, given that synaptic transmission at the level of the photoreceptor–bipolar cells–horizontal cell triad is extremely sensitive to changes in pH [35,39]. Finally, the effects observed by the action of Glycine and its antagonist, Strychnine, could also be perfectly explained by its action on the glycinergic receptors expressed by the axon terminal of the rod bipolar cells (Figure 4). Although a lower amplitude of the oscillatory responses could be expected due to the effect of Glycine, it has been observed that its action is very transient [34], so that a complete abolition of the oscillatory response would not take place. The effect of Strychnine would be very similar to Bicuculline. Although the reciprocal synapse between rod bipolar cells and AII amacrine cells is blocked, the functionality of the reciprocal synapses between rod bipolar cells and A17 amacrine cells would be maintained, which, acting on transient GABA_A_ receptors, would at least partially maintain the oscillations.

Based on the above-mentioned explanations, we propose that reciprocal synapses between glutamatergic rod bipolar cells and glycinergic AII amacrine cells or between rod bipolar cells and GABAergic A17 amacrine cells would explain the generation of OPs under dark-adapted conditions. However, the physiological significance of these reciprocal synapses remains to be definitively elucidated; although, they may contribute to the lateral interaction between bipolar rod cells. Further experiments will need to be performed at the cellular level to address these mechanisms. Finally, we cannot discard the possibility that the reciprocal synapses between OFF-cone bipolar cells and AII Amacrine cells may somehow contribute to OP oscillations, since ERG-OPs recorded from human beings affected by achromatopsia suffer a decrease in the magnitude of the OPs [40].

## 4. Materials and Methods

### 4.1. Animal Model, Legal Protection, and Maintenance

Healthy male and female adult mice of the wild-type strain C57BL/6J were used. A total of 58 mice were used. The mice were housed in ventilated racks with cages under standard conditions and with circadian cycles of 12/12 h of light–darkness, and free access to diet and water. The maintenance temperature was 21 ± 1 °C, the relative humidity of the air was 55 ± 10%, and the ventilation was >30 changes/hour. All experimental procedures followed Directive 2012/63/EU of the European Parliament and of the Council, and RD 53/2013 of the Spanish Regulation for the protection of animals used for scientific purposes and were approved by the Committee of the Community of Madrid for the use of laboratory animals (Proex 143/17).

### 4.2. Animal Preparation and Intravitreal Drug Injection

Dark-adapted animals (>12 h completely darkness) were anesthetized with an intraperitoneal injection of 0.5 mL/150 gr saline solution (0.9% NaCl) containing ketamine (100 mg/Kg) (Ketamidor, Laboratorios Karizoo, S.A. Barcelona, Spain) and xylazine (Xilagesic, Laboratorios Calier, S.A. Barcelona, Spain) (5 mg/Kg). Subsequently, right eye of each animal was punctured just behind the limbus using a Hamilton microsyringe (Hamilton Company, Reno, NV, USA) with 34G needle under dim red-light illumination. In the control group (*n* = 6), the animal’s right eye (RE) was punctured with 1 μL of PBS while the left eye (LE) was not manipulated. In the experimental groups, the LE was injected with 1 μL of PBS solution as a control, and the RE of each animal was injected with 1 μL of a PBS solution containing one of the following drugs, which are agonists or antagonists of retinal neurotransmitters: APB (2-amino-4phosphonobutyric acid, 25 mM), DNQX (6,7-dinitroquinoxaline-2,3-dione, 30 mM), Glutamate (100 mM), Bicuculline (10 mM), GABA (γ-Aminobutyric acid, 100 mM), TPMPA (1,2,5,6-tetrahydropyridine-4-yl methylphosphinic acid, 25 mM), Glycine (10 mM), Strychnine (25 mM), or HEPES (4-(2-hydroxyethyl)-1-piperazineethanesulfonic acid, 25 mM), into a final mouse vitreous volume of 5 μL [41]. The anesthesia and intraocular injections were carried out under dim red-room illumination. Unless otherwise indicated, all drugs were purchased from Sigma Aldrich (St. Louis, MO, USA). Drugs and reagents were prepared fresh in PBS. Each animal was used for one single drug application.

Before recordings, pupils were dilated with one drop of 1% tropicamide (Colircusí tropicamide, Alcon Cusí, Barcelona, Spain). Animals were then placed at the center of a homemade Ganzfeld dome for full-field ERG. The body temperature of the animal was maintained at 37 °C by using a water-circulation warming pad. To preserve the corneal surface from desiccation and facilitate the transmission of the electrical signals, a drop of 2% methyl-cellulose (Methocel, Omnivision, Puchheim, Germany) was applied on the corneal surface. Mice were then kept for 10 min in complete darkness to let drugs work efficiently and preserve the animals’ full dark adaptation.

### 4.3. Signal Recording and Light Stimulation

For OP recording, Burian–Allen corneal electrodes were placed on the visual axis, at 2–3 mm from the cornea; reference electrode was carefully placed on the mouth preventing the mouse from swallowing the tongue. Ground needle electrode was placed in the base of the tail. All electrodes were connected to an AC amplifier (Grass^®^, Astro-Med Inc., West Warwick, Rhode Island, USA); electrophysiological data acquisition and analysis system (Power-Lab-ADI^®^ and Labchart^®^ 8 software, Oxford, UK), were used for signal acquisition and storage, since ERG a- and b-waves, which consist of lower frequency components (20–50 Hz), appear as the initial big negative wavelet in the OP response [20]. Thus, the settled high-pass filter for OP recording was 50 Hz, the low-pass filter was 1000 Hz, and the digitized sample rate was at 2 kHz, which was higher than 1 kHz for faithful capture of OPs’ signal [22]. Meanwhile, there were 100 milliseconds of pre-stimulation. To better observe the OP response, sometimes ERG response was also recorded during the same OP recording session but changed the parameters of the filter, having a high-pass filter of 0.1 Hz and a low-pass filter of 1000 Hz. All the recorded electrophysiological responses were amplified (×1000).

White light stimuli were derived from an LED-based homemade Ganzfeld dome. Light intensities were measured with a calibrated photometer (Gossen Mavo Monitor, Germany) and based on the full-field flash ERG standards [2], adapted for mouse recordings. Under dark adaptation (scotopic condition), the rod-driven response corresponded to 0.01 cd·s·m^−2^, and the rod/cone-driven mixed response to 15 cd·s·m^−2^, at interstimulus intervals of 5 s and 15 s for preserving the animals’ dark adaptation status, respectively. Under light adaptation, the cone-driven response (photopic condition) corresponded to 15 cd·s·m^−2^ at an interstimulus interval of 1 s. The experiments were conducted in a silent room. The recording started from rod-driven and continued with rod/cone-driven mixed response. After that, mice experienced 15 min of light adaptation under background light of 32 cd·m^−2^, which saturated the rod-driven responses. Then, the cone-driven response was recorded. To acquire stable responses, a total of at least 30 light responses were averaged for each animal in response to each light intensity by reducing the impact of noise and artifacts. Once ending the experiment, the mice were sacrificed with sodium pentobarbital (Doletal^®^, Vetoqimol, Madrid, Spain).

### 4.4. OP Analysis

The OP waveforms and response amplitudes were analyzed offline. Frequency analyses were performed after high-pass 100 Hz digital filtering was applied to the recorded traces. The Fast Fourier Transform was calculated by the Labchart^®^ software for each 100 Hz filtered recording trace. Data of the peak power density (μV^2^/Hz) from different animal groups were averaged. Data are reported as mean and SD (standard deviation). Normality of the data was verified by Kolmogórov–Smirnov test. Statistical significance was assessed with Student’s t-test analyses by IBM SPSS statistical 23.0 package (SPSS Inc., Chicago, IL, USA) for paired responses. *p* values of less than 0.05 were considered statistically significant.

## 5. Conclusions

We propose that reciprocal synapses between glutamatergic rod bipolar cells and glycinergic AII amacrine cells and reciprocal synapses between rod bipolar cells and GABAergic A17 amacrine cells are the main mechanisms undelaying the Oscillatory Potentials of the Electroretinogram recorded under a dark adaptation in mice.

## Figures and Tables

**Table 1:** Peak values of Power Density (µV²/Hz) averaged from OP recorded from a series of animals injected with different agents. All OP traces were recorded under dark adaptation to light stimuli of 15 cd·s·m^−2^.

	APB		GABA		Bicuculline		TPMPA		Glutamate		DNQX		Glycine		Strychnine		HEPES	
	(25 mM)		(100 mM)		(10 mM)		(25 mM)		(100 mM)		(30 mM)		(10 mM)		(25 mM)		(25 mM)	
	LE	RE	LE	RE	LE	RE	LE	RE	LE	RE	LE	RE	LE	RE	LE	RE	LE	RE
mean	4.10	0.00	4.67	0.00	4.26	2.30	4.10	4.03	4.06	0.00	3.90	0.00	3.94	1.88	4.50	2.00	4.28	1.63
SD	0.28	0.00	0.88	0.00	0.33	0.61	0.28	0.21	0.33	0.00	0.28	0.00	0.17	0.45	0.85	1.05	0.57	1.12
*n*	6		6		6		6		6		6		4		6		4	
*p*	<0.001		<0.001		<0.001		0.285		<0.001		<0.001		0.003		<0.001		0.023	

LE: Left Eye; RE: Right Eye; *n*: number of animals; *p*: statistical significance—Student’s *t*-test.

## Data Availability

Not applicable.
